# A Prospective Randomized Controlled Trial of the Effect of Maintenance of Continuous Cuff Pressures (20 cmH2O vs 30 cmH2O) on Postoperative Airway Symptoms in Laparoscopic Surgeries

**DOI:** 10.7759/cureus.47816

**Published:** 2023-10-27

**Authors:** Muraki Mami, Mitsutaka Edanaga, Haruka Mizuguchi, Miyuki Sugimoto, Shuji Yamamoto, Michiaki Yamakage

**Affiliations:** 1 Department of Anesthesiology, Sapporo Medical University, Sapporo, JPN; 2 Department of Anesthesiology, Otaru General Hospital, Otaru, JPN; 3 Department of Anesthesiology, Obihiro Kosei General Hospital, Obihiro, JPN

**Keywords:** smartcuff, laparoscopic surgery, endotracheal tube cuff pressure monitoring, postoperative hoarseness, postoperative sore throat

## Abstract

Introduction: Recently, laparoscopic surgery has been used in many fields of surgery. It has been reported that cuff pressure becomes high during laparoscopic surgery. Increased cuff pressure may cause postoperative sore throat and hoarseness. Considering previous reports, we hypothesized that maintenance of a fixed low cuff pressure during laparoscopic surgery might be associated with low grades of postoperative sore throat and hoarseness.

Methods: The participants were 100 patients between 20 and 80 years of age who were scheduled to undergo laparoscopic surgery lasting over 2 hours. Patients were randomly allocated to two groups with endotracheal tube cuff pressures fixed at 20 cmH2O (low-pressure group; LPG) and 30 cmH2O (high-pressure group; HPG). We evaluated mainly sore throat and hoarseness on postoperative day 1 using a visual analog scale (VAS; 0-10 cm). Statistical comparisons of values were performed using the unpaired t-test, Mann-Whitney U-test, and chi-square test with values of p < 0.05 considered statistically significant.

Results: There were no significant differences in background characteristics between the two groups. Median postoperative scores for the LPG and HPG were 1 (interquartile range, 0-3) and 0 (0-2; p = 0.560) for sore throat and 2 (0-4) and 1 (0-3; p = 0.311) for hoarseness, respectively, and the differences were not significant.

Conclusion: The effects of maintenance of a fixed low cuff pressure and a fixed high cuff pressure on the degrees of postoperative sore throat and hoarseness after laparoscopic surgery were the same and the grades were low.

## Introduction

Recently, laparoscopic surgery and robotic surgery have been used in many fields of surgery because of their minimal invasiveness for patients. It was reported that the duration of laparoscopic surgery tended to be longer than that of open surgery [1,2]. Thus, patients scheduled for laparoscopic or robotic surgery would need a long duration of artificial ventilation using an endotracheal tube. According to previous studies, the cuff pressure of an endotracheal tube is often high during laparoscopic surgery because of pneumoperitoneum [3,4]. In addition, the increased peak and mean airway pressures would be associated with increases in cuff pressure [3]. The increased cuff pressure may cause postoperative sore throat and hoarseness [5-7]. Smartcuff® (Smiths Medical, Tokyo, Japan) has been used in Japan since 2018 for maintaining a fixed cuff pressure. This device can automatically adjust the cuff pressure to the set cuff pressure when it is connected to the cuff, and it continues to measure and adjust the cuff pressure as long as the device is connected. Therefore, the cuff pressure remains constantly adjusted during the surgery regardless of the patient's position and pneumoperitoneum. Although there has been one study on the efficacy of automatic retention pressure with a double-lumen tube cuff, the effects of fixed cuff pressure on sore throat and hoarseness after laparoscopic surgery have not been examined in any previous studies [8].

In the postoperative intensive care field, cuff pressure of more than 20 cmH2O has been recommended for preventing ventilation-induced pneumonia [9]. On the other hand, cuff pressure of less than 30 cmH2O has been recommended for preventing tracheal injury [10,11]. As mentioned above, a cuff pressure of 20-30 cmH2O is recommended during intubation. Until now, the cuff pressure has been adjusted only once after intubation, and even after adjusting to the recommended cuff pressure, the cuff pressure could change during surgery. No study has yet compared the effects on postoperative sore throat and hoarseness by maintaining cuff pressure continuously during surgery at 20 cmH2O, the lowest recommended cuff pressure, and at 30 cmH2O, the highest recommended pressure. We hypothesized that maintenance of a fixed low cuff pressure (20 cmH2O) during laparoscopic surgery might be associated with low grades of postoperative sore throat and hoarseness compared with the effects of a fixed high cuff pressure (30 cmH2O).

## Materials and methods

Subjects and study design

This prospective, single-blinded, randomized controlled trial (302-224) was approved by the institutional review board of Sapporo Medical University, Hokkaido, Japan on March 14, 2019, and was registered in the UMIN clinical registry (ID: UMIN000036388) before patient enrollment. After providing written informed consent, patients who were scheduled to undergo laparoscopic surgery were enrolled from April 2019 to January 2020. A total of 100 patients between 20 and 80 years of age who were scheduled to undergo laparoscopic surgery that was predicted to last for more than 2 hours were enrolled. Patients taking tranexamic acid, which has an inhibitory effect on sore throat, patients taking antitussives, patients who had dementia, and patients who were unable to cooperate were excluded. Participants for whom surgery was completed in less than 2 hours were also excluded.

Randomization

We used a parallel design with an allocation ratio of 1:1, and data were collected from Sapporo Medical University School of Medicine, Sapporo, Japan, and Obihiro Kosei General Hospital, Obihiro, Hokkaido, Japan. Block randomization was used. Subjects were randomly allocated to two groups with endotracheal tube cuff pressure fixed to either 20 cmH2O as a low-pressure group (LPG) or 30 cmH2O as a high-pressure group (HPG). Attending anesthesiologists knew the group assignments before surgery, but the subjects were blinded to allocations.

Anesthesia

Intubation was conducted by anesthesiologists. The method for anesthesia induction was rapid induction for all participants. For induction, propofol, rocuronium, and remifentanil were used. Although nitrous oxide was not used in this study, the agents used for the maintenance of anesthesia, whether total intravenous anesthesia or inhaled anesthesia, were decided by each anesthesiologist. A McGRATH™MAC (Medtronic, Tokyo, Japan) laryngoscope was used for all cases, and Parker endotracheal tubes with internal diameters of 6.5 mm for females and 7.5 mm for males were used. In a previous study, the use of an endotracheal tube with an internal diameter of 7.0 mm was shown to be correlated with higher risks for postoperative sore throat and hoarseness compared to an endotracheal tube with an internal diameter of 6.0 mm in women [12]. To avoid the bias for sore throat and hoarseness, we decided to use a tube size that is smaller than the size we usually use in a clinical situation. In both groups, fixed cuff pressure was set immediately after intubation and continuously monitored and titrated to each cuff pressure using Smartcuff®. The cuff was filled with air. 

Data collection

Data were collected on postoperative day 1 by anesthesiologists. Patients evaluated the degree of postoperative sore throat and hoarseness using a visual analog scale (VAS; 0-10 cm) with 0 cm indicating no sore throat or hoarseness and 10 cm indicating the most severe sore throat or hoarseness that the patient could imagine. Collected data were recorded on an electronic health record.

Statistical analyses and outcome variables 

Data for the following items were collected for all patients: age, weight, height, sex, smoking history, preoperative diagnosis, surgical type, number of intubation attempts, duration of surgery, duration of anesthesia, type of inhaled anesthetics, amount of fentanyl, amount of remifentanil and postoperative complications other than sore throat or hoarseness. We evaluated mainly the degree of sore throat and hoarseness on postoperative day 1 using the VAS. We also examined the relative risks and odds ratios (ORs) of gender and smoking for sore throat and hoarseness. Statistical analysis was performed using GraphPad Prism version 6 software (MDF Co., Tokyo, Japan). Age, weight, height, duration of the operation, duration of anesthesia, amount of fentanyl, and amount of remifentanil are presented as means ± SD and were analyzed using the unpaired t-test. Incidences of postoperative sore throat and hoarseness were also analyzed using the unpaired t-test. The degrees of postoperative sore throat and hoarseness are presented as medians and interquartile ranges and were compared using the Mann-Whitney U-test. Gender, smoking history, and type of inhaled anesthetics were analyzed by the chi-square test. Relative risks and ORs of gender and smoking history were analyzed using Fisher’s exact test. Values of p < 0.05 were considered statistically significant. There was no previous study on a comparison of airway symptoms with different fixed cuff pressures during laparoscopic surgery. We therefore considered the necessary sample size based on a past report on a comparison of inflation pressures in endotracheal tube cuffs [13]. According to that report about cuff pressures, the incidences of postoperative sore throat were 54% in the control group and 12% in the study group. Considering the same difference, the minimum number of patients needed for each group was 27 with an alpha error of 0.05 and a study power of 0.9. Considering a possible dropout rate of 10%, 30 patients per group as the minimum number of patients were included. By referring to that study, we decided on a sample size of 100 cases.

## Results

**Figure 1:**
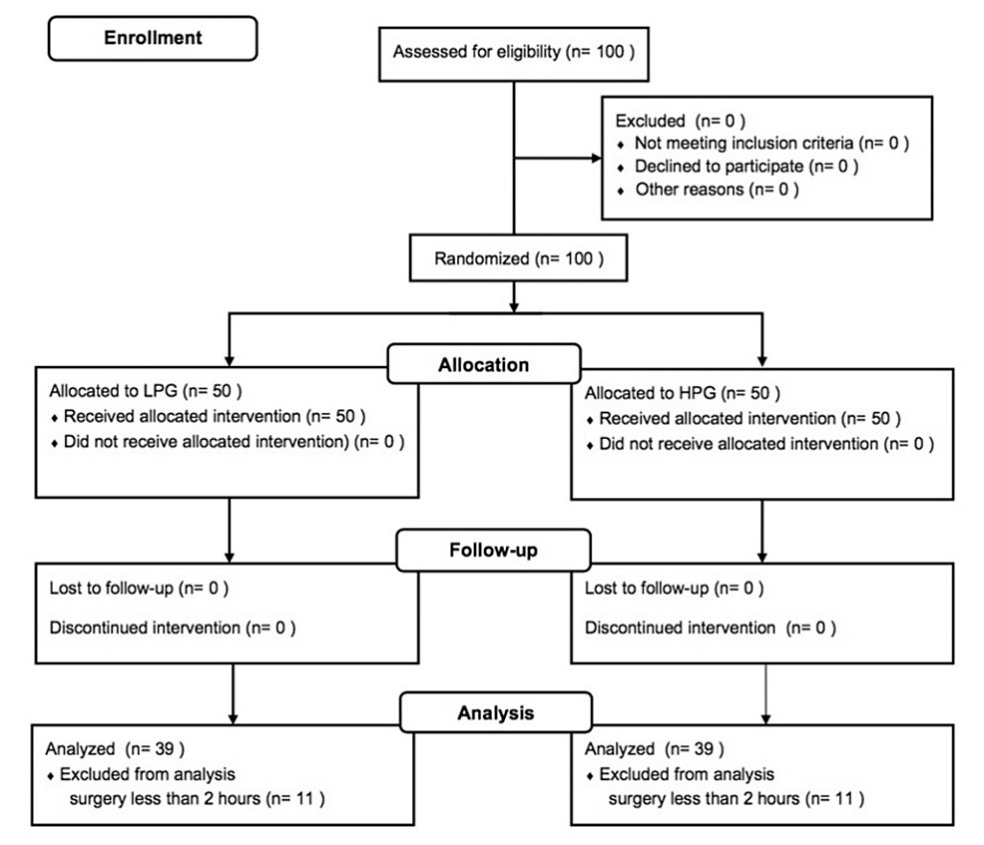
A CONSORT flow diagram showing participant flow through each stage of the randomized controlled trial LPG: low-pressure group; HPG: high-pressure group

From April 10, 2019, to January 8, 2020, we enrolled 100 patients (72 patients at Sapporo Medical University and 28 patients at Obihiro Kosei General Hospital). Eleven patients in the LPG and 11 patients in the HPG were excluded from the final analysis because the duration of surgery was less than the planned minimum duration of 2 hours. No study protocol violation was reported during the study period. Thus, data for a total of 78 patients (39 patients in each group) were used for statistical analysis (Figure 1).

In all cases, intubation was successful on the first attempt using McGRATH™MAC. Patient characteristics are shown in Table 1.

**Table 1:** Baseline patient characteristics LPG: low-pressure group; HPG: high-pressure group; CI: confidence interval Values represent means ± SD or numbers (percentage)

	LPG	HPG	P value	95% CI
N	39	39		
Age (years)	57 ± 14	62 ± 12	0.078	
Height (cm)	163 ± 9	160 ± 9	0.066	
Weight (kg)	65 ± 14	60 ± 13	0.107	
Gender, male : female	19 : 20	17 : 22	0.650	
Smoking (%)	22 (56)	24 (62)	0.645	
Tracheal tube size (6.5 mm : 7.5 mm)	20 : 19	22 : 17		
Duration of surgery (min)	250 ± 100	269 ± 134	0.478	-34.77 to 73.54
Duration of anesthesia (min)	322 ± 116	352 ± 158	0.362	-34.17 to 92.53
Number of attempts of intubation	1	1	1.0	
Surgery				
General surgery	11 (28)	17 (44)		
Gynecologic surgery	13 (33)	15 (38)		
Urologic surgery	14 (36)	7 (18)		
Others	1 (2)	0		
Type of inhaled anesthetics Sevoflurane vs desflurane	14 vs 15	9 vs 27	0.069	
Amount of fentanyl (mg)	379 ± 272	387 ± 243	0.887	-108.0 to 124.6
Amount of remifentanil (mg)	2.0 ± 1.1	1.8 ± 0.9	0.249	-0.7451 to 0.1964

**Figure 2:**
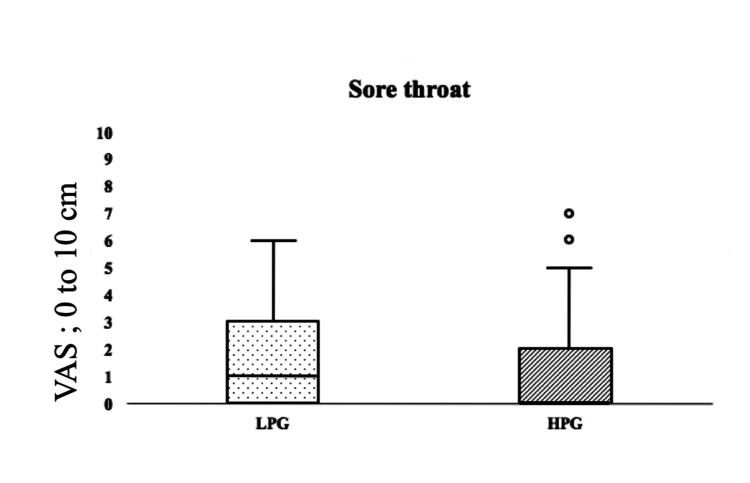
VAS score of postoperative sore throat LPG: low-pressure group (20 cmH2O); HPG: high-pressure group (30 cmH2O); VAS: visual analog scale * p < 0.05 versus LPG. Degrees of postoperative sore throat in the LPG (black spots) and HPG (black slanted lines) are shown as medians {interquartile range}. There was no significant difference between the two groups.

There were no significant differences between the two groups in the characteristics (age, height, weight, type of inhaled anesthetics, duration of surgery, and duration of anesthesia). Chi-square testing of gender (p = 0.650), smoking history (p = 0.645), amount of fentanyl (p=0.887), and amount of remifentanil (p=0.249) showed no significant differences. The degrees of sore throat on postoperative day 1 for the LPG and HPG were 1 {IQR, 0-3} and 0 {IQR, 0-2} (p = 0.560), respectively, and the difference was not significant (Table 2 and Figure 2).

**Table 2:** Postoperative sore throat and hoarseness LPG: low-pressure group; HPG: high-pressure group Values represent medians {interquartile range} or numbers (percentage)

	LPG	HPG	P value
Degree of sore throat	1 {0–3}	0 {0–2}	0.560
Degree of hoarseness	2 {0–4}	1 {0–3}	0.311
Incidence of sore throat (%)	23 (59)	18 (46)	0.263
Incidence of hoarseness (%)	27 (69)	23 (59)	0.761

**Figure 3:**
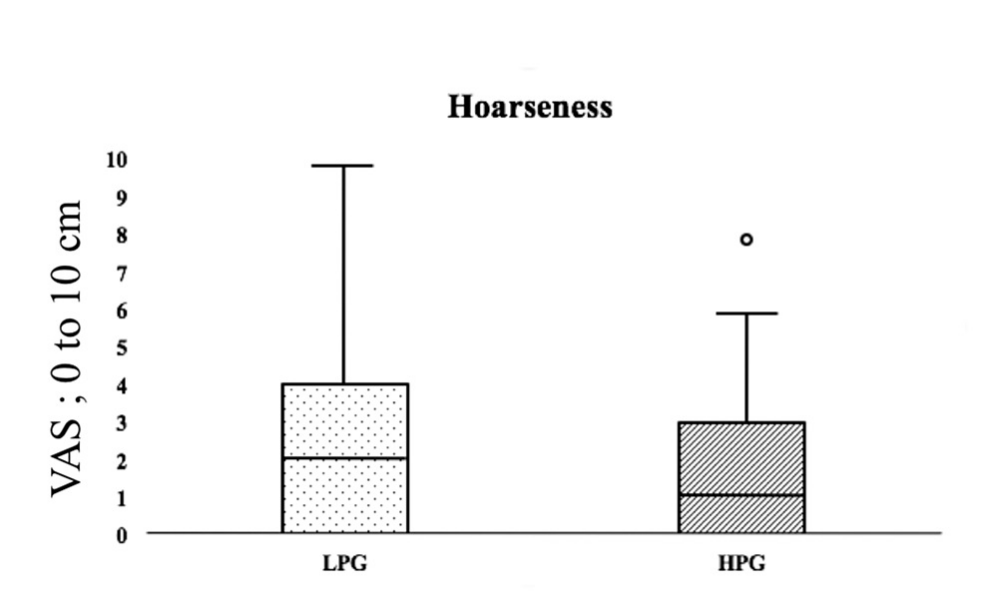
VAS score of postoperative hoarseness LPG: low-pressure group (20 cmH2O); HPG: high-pressure group (30 cmH2O); VAS: visual analog scale * p < 0.05 versus LPG. Degrees of postoperative hoarseness in the LPG (black spots) and HPG (black slanted lines) are shown as medians {interquartile range}. There was no significant difference between the two groups.

The degrees of hoarseness on postoperative day 1 for the LPG and HPG were 2 {IQR, 0-4} and 1 {IQR, 0-3} (p = 0.311), respectively, and the difference was not significant (Table 2 and Figure 3).

Also, the incidences of postoperative sore throat (59% vs 46%, p = 0.263) and hoarseness (69% vs 59%, p = 0.761) were not significantly different between the two groups (Table 2). Risk factors for postoperative sore throat and hoarseness are shown in Table 3 and Table 4.

**Table 3:** Risk factors for postoperative sore throat OR: odds ratio; CI: confidence interval Data were analyzed using Fisher’s exact test

	OR	95% CI	P value
Gender (female to male)	1.211	0.496–2.955	0.820
Smoking history (smoking to no-smoking)	1.473	0.595–3.649	0.491

**Table 4:** Risk factors for postoperative hoarseness OR: odds ratio; CI: confidence interval Data were analyzed using Fisher’s exact test

	OR	95% CI	P value
Gender (female to male)	1.273	0.503–3.219	0.643
Smoking history (smoking to no-smoking)	0.707	0.272–1.835	0.632

Gender (female to male) and smoking history (smoking to non-smoking) did not affect the risk of postoperative sore throat or hoarseness. No other complications associated with tracheal intubation were evident in either group.

## Discussion

This study firstly showed that the effects of maintenance of a fixed high cuff pressure (30 cmH2O) on the degrees of sore throat and hoarseness on postoperative day 1 after laparoscopic surgery were the same as those of a fixed low cuff pressure (20 cmH2O). Although past studies showed that laparoscopic surgery increased cuff pressure and caused airway symptoms, our results obtained by continuously maintaining a fixed cuff pressure (low or high) during laparoscopic surgery may be associated with the low grade of uncomfortable airway symptoms. Studies have shown that the incidence of postoperative sore throat is 14% to 50% and that severe postoperative sore throat can affect the eating behaviors of patients and reduce satisfaction during hospitalization [14,15]. Also, the incidence of postoperative hoarseness has been reported to be 40% to 50% [14,16]. In many past studies, postoperative sore throat and hoarseness were shown to be associated with various factors including age, gender, tube size, number of intubation attempts, cuff pressure, duration of the operation, type of volatile anesthetics, and with or without laparoscopic surgery [3,4,16-18]. First, considering the factor of age, women over 40 years of age were shown to be at greater risk for postoperative sore throat [12]. In our study, 68 (87%) of the 78 patients were older than 40 years of age. The factor of age > 40 years in our study might be not a risk factor for sore throat. It has also been reported that the frequency of sore throat after tracheal intubation in women is two times higher than that in men [16]. The incidences of postoperative sore throat in women (55%) and men (50%) in our study are different from the results for women (37%) and men (32%) in previous studies [12,17]. Those previous studies included patients who underwent general, orthopedic, urological, gynecological, plastic, and ear-nose-throat surgeries and patients who underwent hand surgery and eye surgery, while our study focused on patients who underwent laparoscopic surgery. As mentioned above, laparoscopic surgery would increase the cuff pressure of the endotracheal tube intraoperatively. In addition, the Trendelenburg position in laparoscopic surgery may affect cuff pressure. However, the degree of sore throat on postoperative day 1 was low in our study. This result would be due to the maintenance of a fixed cuff pressure.

In this study, we used a 6.5 mm tracheal tube for women and a 7.5 mm tracheal tube for men. In general, tracheal tubes of 7.0 mm for women and 8.0 mm for men are used in clinical situations. However, a 7.0 mm tube size for women showed a high OR for postoperative hoarseness in a previous study compared to a 6.0 mm tube size [12]. We decided on the tracheal tube size by considering the bias of postoperative hoarseness. The incidences of postoperative hoarseness were 67% in women and 64% in men in our study. However, the degrees of postoperative hoarseness on postoperative day 1 in the two groups were low. As shown in Table 3 and Table 4, statistical analysis indicated that gender (female to male) showed no elevated relative risk for postoperative sore throat or hoarseness. Therefore, our study indicates that a continuous fixed high cuff pressure (30 cmH2O) is appropriate as is a low cuff pressure (20 cmH2O) during laparoscopic surgery.

Recently, a prospective study showed that the incidence of postoperative sore throat in patients in whom sevoflurane was used during the operation was lower than that in patients in whom desflurane was used [18]. The reason for that difference may be irritability and inflammation of the airway tract caused by desflurane [19]. In this study, desflurane tended to be used more in the HPG than in the LPG. However, the incidence of postoperative sore throat was different from that in the previous study. As mentioned above, it is thought that the cause of postoperative sore throat is multifactorial.

We used McGRATH™MAC to reduce irritation to the respiratory tract mucosa during tracheal intubation. No other complications such as arytenoid cartilage dislocation occurred in any of the patients. Blind intubation and lack of certainty regarding exact placement may cause mucosal irritation and edema. In a past study, patients in whom Glidescope® (Verathon Medical, Bothell, WA, USA) was used had a low incidence of postoperative sore throat compared with that in patients in whom Pentax AirwayScope (Pentax AWS; Pentax, Tokyo, Japan) was used [20]. In another study, tracheal intubation by McGRATH™MAC with a stylet was compared with that by McGRATH™MAC without a stylet, and there were no significant differences in the incidence of postoperative sore throat, degree of sore throat, and incidence of postoperative hoarseness [21]. McGRATH™MAC with a stylet was used in all of the patients in our study. Postoperative sore throat scores in the LPG and HPG in our study were lower than those in the previous study using McGRATH™MAC with a stylet (score of 5 {IQR, 3-6}) and McGRATH™MAC without a stylet (score of 5 {IQR, 3-6}). A careful procedure for intubation and maintenance of a fixed cuff pressure is important for reducing the incidences of postoperative sore throat and hoarseness.

Some limitations to this study merit consideration. First, we did not consider the effects of position during the operation. The Trendelenburg position used during laparoscopy has been reported to increase airway pressure and endotracheal cuff pressure [22]. Although there was no change in cuff pressure related to the position in our study, the increased airway pressure might affect postoperative sore throat and hoarseness due to edema of the tracheal mucosa. Next, we asked patients about the degree of sore throat and hoarseness on postoperative day 1, but time points (such as 2, 4, and 6 hours) after the operation were not set as outcomes. This might have affected the degrees of postoperative sore throat and hoarseness. Also, we did not control or standardize the anesthesia protocol. The depth of anesthesia during intubation and extubation, the use of analgesia, and the use of a suction catheter were dependent on each anesthesiologist. Finally, we did not compare similar cuff pressures and randomize into a fixed cuff pressure versus a variable cuff pressure. We also did not maintain the cuff pressure above 30 cmH2O due to ethical considerations and selected a cuff pressure of 30 cmH2O for HPG, which is within the recommended range of pressures. Therefore, future studies are needed to evaluate the effects of maintaining the continuously fixed cuff pressure higher than 30 cmH2O.

## Conclusions

In conclusion, our analysis showed that the effects of maintenance of a fixed high cuff pressure (30 cmH2O) on the degrees of sore throat and hoarseness on postoperative day 1 after laparoscopic surgery were the same as those of a fixed low cuff pressure (20 cmH2O). The maintenance of a fixed cuff pressure (20 or 30 cmH2O) is associated with a low degree of sore throat or hoarseness and the use of Smartcuff® makes it possible to maintain these cuff pressures without being affected by the body position or pneumoperitoneum. Since the method for anesthesia and surgical technique were not standardized in this study, further research is needed.
